# Elevated aerosol layer over South Asia worsens the Indian droughts

**DOI:** 10.1038/s41598-019-46704-9

**Published:** 2019-07-16

**Authors:** Suvarna Fadnavis, T. P. Sabin, Chaitri Roy, Matthew Rowlinson, Alexandru Rap, Jean-Paul Vernier, Christopher E. Sioris

**Affiliations:** 1 Indian Institute of Tropical Meteorology, Pune, India; 2 School of Earth and Environment, University of Leeds, Leeds, UK; 3 National Institute of Aerospace, Hampton, Virginia United States; 4 NASA Langley Research Center, Hampton, Virginia United States; 5 Air Quality Research Division, Environment and Climate Change, Toronto, Canada

**Keywords:** Atmospheric science, Atmospheric chemistry

## Abstract

Droughts have become more severe and recurrent over the Indian sub-continent during the second half of the twentieth century, leading to more severe hydro-climatic and socio-economic impacts over one of the most densely populated parts of the world. So far, droughts have mostly been connected to circulation changes concomitant with the abnormal warming over the Pacific Ocean, prevalently known as “El Niño”. Here, exploiting observational data sets and a series of dedicated sensitivity experiments, we show that the severity of droughts during El Niño is amplified (17%) by changes in aerosols. The model experiments simulate the transport of boundary layer aerosols from South Asian countries to higher altitudes (12–18 km) where they form the Asian Tropopause Aerosol Layer (ATAL) (~ 60–120°E, 20–40°N). During El Niño, the anomalous overturning circulation from the East Asian region further enriches the thickness of aerosol layers in the ATAL over the northern part of South Asia. The anomalous aerosol loading in the ATAL reduces insolation over the monsoon region, thereby exacerbating the severity of drought by further weakening the monsoon circulation. Future increases in industrial emissions from both East and South Asia will lead to a wider and thicker elevated aerosol layer in the upper troposphere, potentially amplifying the severity of droughts.

## Introduction

The century-long systematic monitoring of Indian monsoon records reveals that frequent floods and droughts were not unfamiliar to this nation of 1.3 billion people. Statistical analysis shows that in the second half of the twentieth century the frequency of droughts has increased relative to the first half^1,2^. A number of studies attribute droughts to El Niño effects^3–5^, which modify the Walker and Hadley large-scale circulations through atmospheric tele-connections. The existing literature shows that most of the Indian droughts are accompanied by El-Niños^5–7^. In addition to the increasing frequency of droughts, South Asia underwent a widespread declining trend in its total seasonal monsoon precipitation (~7% from 1951 to the present). This is largely blamed on the anthropogenic aerosols as they generally reduce temperatures over land, resulting in weaker land-sea thermal contrast^2,8,9^. The land-sea thermal contrast is the essential force required to pull the monsoon circulation from ocean to land. Aerosols interact in many ways with the monsoon - the enhancement or suppression of the Indian summer monsoon rainfall depends on its duration and scale (including remote influences), along with its distinct direct and indirect radiative effects^8–10^.

In addition to this conventional understanding, the presence of the recently discovered aerosol layer in the upper troposphere and lower stratosphere (UTLS) known as “the Asian Tropopause Aerosol Layer (ATAL)” covering South Asia (defined here as the region spanning 0–100°E, 20–45°N) during the monsoon season^11^ may have a role in controlling the monsoon precipitation. The development of the ATAL is associated with convective transport of aerosols from the lower atmosphere to the UTLS^12–14^. Observations from satellites (CALIPSO, SAGE-II), balloon-borne experiments (BATAL)^15^, (StratoClim)^16^, and CARIBIC aircraft (Civil Aircraft for the Regular Investigation of the atmosphere Based on an Instrument Container – Lufthansa, Airbus 340–600) reveal that the ATAL extends over the 12–18 km range and is possibly composed by sulfate aerosols along with black-carbon, organic aerosols, nitrates and dust particles^17,18^. The unprecedented growth in Asian aerosol emissions during the past few decades, mainly from China and India, is therefore expected to exacerbate this phenomenon^8,19^. Indian emissions have an important contribution to the ATAL due to the vicinity of the centre of Asian monsoon convective updraft^20^. This region shows a higher growth in sulfate aerosol compared to China^21^ leading to a pronounced contribution to the ATAL composition. These sulfate aerosols significantly alter the radiative balance of the atmosphere by reflecting incoming solar radiation^22^ back to space. CALIPSO and SAGE-II observations show a positive trend (+0.004 over the period 1996–2014) in UTLS aerosol optical depth during summer which causes a regional radiative forcing of around −0.1 W•m^−2^ over that 18 year period^12^. A previous study^23^ has indicated the presence of large amounts of fine dust particles and carbonaceous aerosols in the upper troposphere in early monsoon season.

Fire-generated aerosol is also commonly observed over Asia during summer months^24,25^, often associated with El Niño events, which promote forest fires over many parts of the globe^26^. For example, widespread forest and peatland fires over maritime southeast Asia in September 2015 released large amounts of aerosols and carbon emissions (carbon monooxide, carbon dioxide, methane)^25,27,28^. The smoke lifted by the monsoon circulation and trapped in the anti-cyclone^13,18^ also alters the regional chemical budget and the associated radiative forcing^12,17^. This usually large contribution from natural sources can mask some of the contribution from anthropogenic aerosol.

Here, using satellite observations and model simulations, we explore for the first time the contribution of anomalously high aerosol loadings over the UTLS region during El Niño in worsening drought conditions over the Indian subcontinent.

## An Added Blanket of Aerosol Over South Asia during El Niño

**Figure 1:**
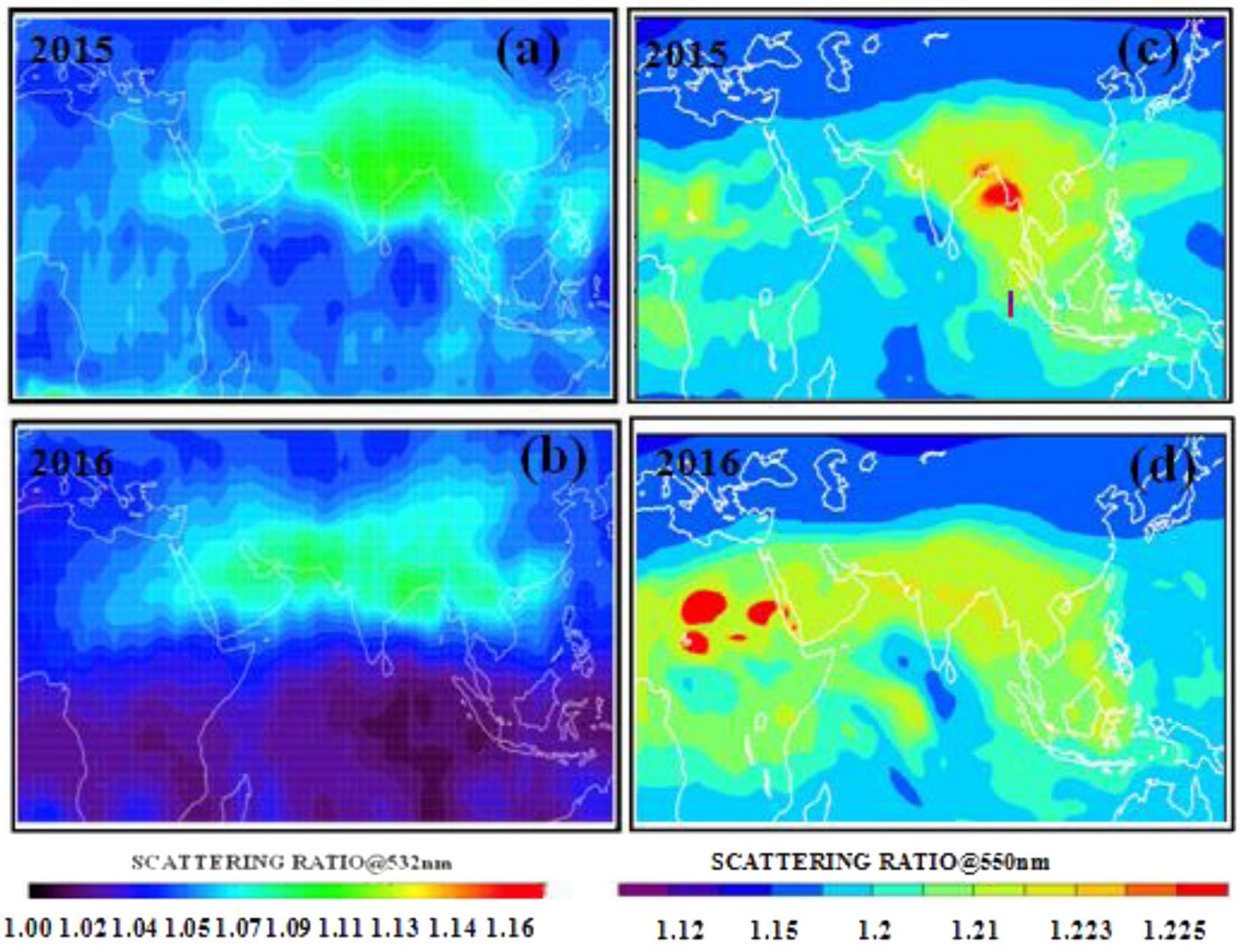
Mean Scattering Ratio (SR) averaged for 15–18 km and for the month of July–August. (**a**) CALIOP observations for 2015 (El Nino year) and, (**b**) CALIOP observations for 2016 (normal year). (**c**) ECHAM5-HAMMOZ simulation (aeronAMIP) for 2015. (**d**) ECHAM5-HAMMOZ simulation (aeronAMIP) for 2016. (Figure created using the MATLAB software).

Observations from the CALIPSO satellite of scattering ratio at 532 nm, a proxy for mixing ratio in the atmosphere, reveal the presence of the elevated layer of aerosol over South Asia during July-August of 2015 and 2016 (Fig. 1a,b). During the El Niño period of July–August 2015, this aerosol layer appears thicker and centered over the entire North Indian monsoon region (15–45°N, 60–120°E), in contrast to the non El Niño period of July–August 2016, when the layer is extended mostly over the Arabian Peninsula (Fig. 1a,b) and less thicker over the monsoon region. The ECHAM6–HAMMOZ simulations reproduce the general observed ATAL features (Fig. 1c,d), although they overestimate the scattering ratio at 550 nm by 0.06–0.11 (details in section 5.4). Importantly, the model simulates the thicker ATAL during the monsoon season of 2015 associated with El Niño conditions over the Pacific. Time series^12^ of CALIPSO scattering ratio at 532 nm (SR@532 nm) averaged over the Asian region (15–45°N, 5–105°E) at 15, 16 and 17 km also peak during the summer of 2009, another El Niño year. However, the 2009 northern hemisphere summer UTLS was also influenced by volcanic aerosols from the Sarychev eruption (Kuril Islands, Russia) in June 2009^29^. While it is difficult to separate volcanic aerosols from ATAL using satellite observations, the use of models allows us to understand the distinct role of ATAL during El Niño years by removing the influence of volcanoes (i.e. the contribution of this eruption on UTLS aerosols is not included in the model). Overall, our model simulations capture the general behavior of the ATAL and the influence of El Niño on its loading and geographic distribution, but have the tendency to overestimate its thickness.

**Figure 2:**
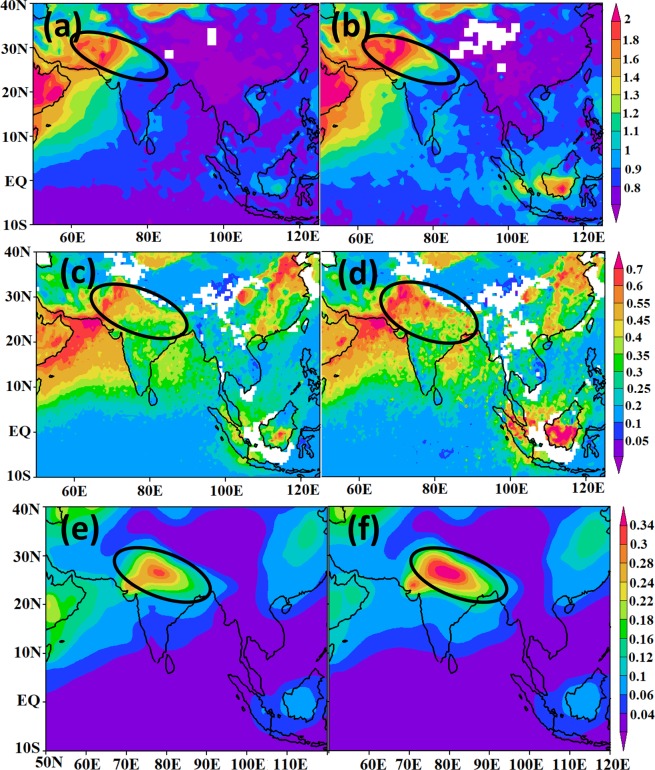
Distribution of seasonal mean (July–September) TOMS aerosols index, aerosol optical depth from MISR and ECHAM5-HAMMOZ model simulation. (**a**) TOMS aerosols index for the non El Niño years during 1978–2005. **(b**) TOMS aerosols index averaged during strong El Niño years considered in this study (1982, 1991, 1994, 1997, 2002, 2004, 2009 and 2015). Aerosol optical depth from MISR for **(c**) non El Niño years during 2000–2013, **(d**) strong El Niño years (2002, 2004, 2009). Distribution of simulated aerosol optical depth (excluding sea salt) at 550 nm as obtained from (**e**) aeronCL, (**f**) aeronEL experiments. In Fig. (**a–e**) the region of elevated aerosol is indicated by the black ellipsoid. (Figure created using the COLA/GrADS software).

The Asian monsoon region exhibits strong convergence from the nearby deserts, the East Asian land mass and adjacent oceans. This makes the monsoon region a preferred destination for many different aerosol species including desert dust, industrial emissions (e.g. sulfate) and local carbonaceous components^19^. Micro pulse lidar at Visakhapatnam and boundary layer lidar observations at Gadanki and Tirupati in India also show elevated aerosol in the lower troposphere (2–5 km) during the monsoon season due to transport from West Asia^30–32^. The monsoon convection can lift these Asian boundary layer aerosols and trace gases to the UTLS^13,18^. Due to the reduced rain and associated scavenging, the aerosol loading over the Indo-Gangetic Plain and North India is anomalously high during El Niño^4,33^ (Fig. 2). Irrespective of the weak upward motion over South Asia, the aerosol loading becomes deeper due to the anomalous inflow of the East Asian aerosols towards the monsoon region which are transported to the upper troposphere over Asia^34,35^.

**Figure 3:**
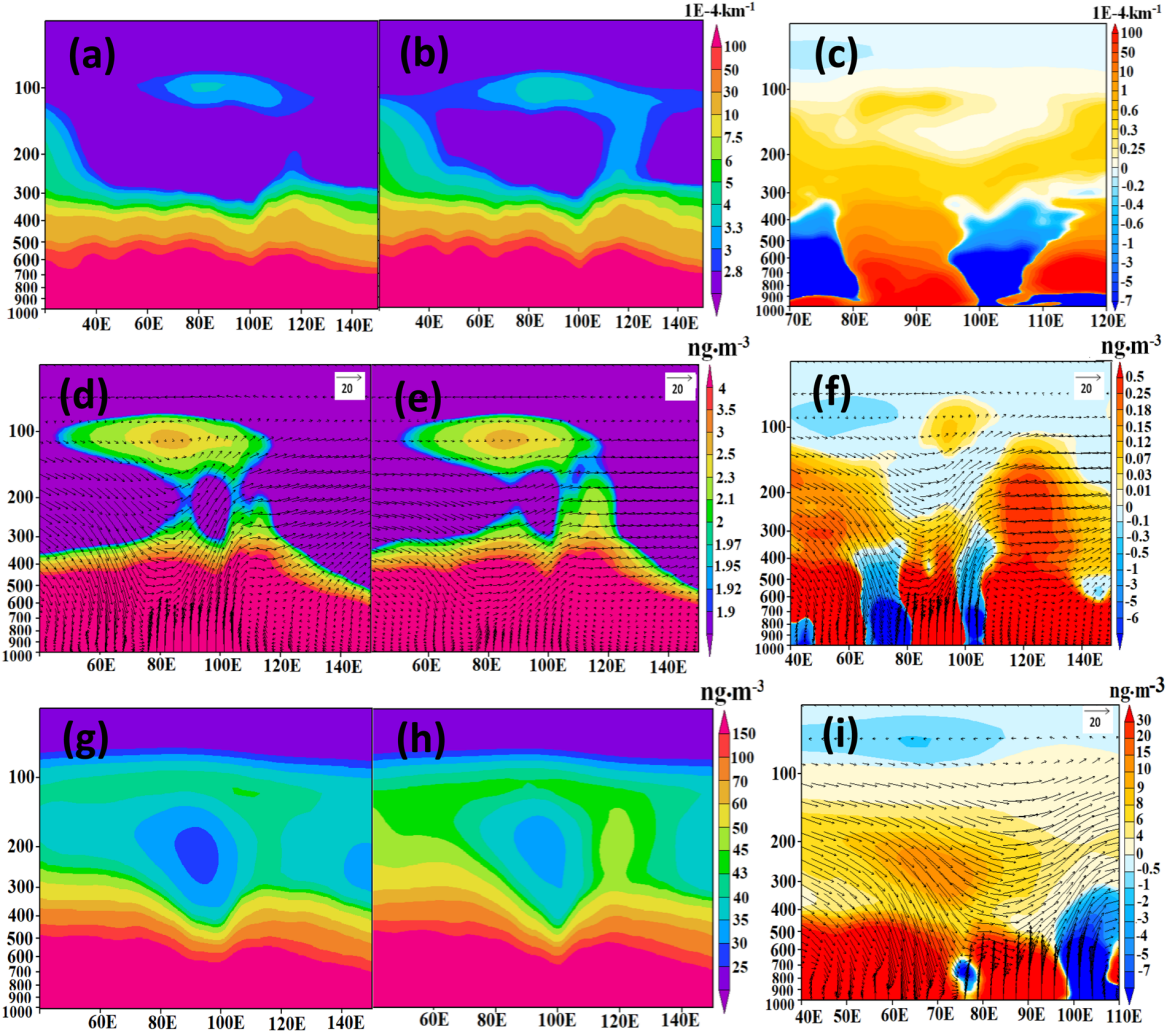
Distribution of seasonal mean (July–September) aerosol extinction (1E-4 km^−1^) zonal cross section (23–40°N) from ECHAM5-HAMMOZ model simulations (**a**) aeronCL, (**b**) aeronEL, (**c**) difference between aeronEL and aeronCL, (**d**–**f**) same as (**a**–**c**) but for black carbon aerosols (ng•m^−3^), (**g–**i) same as (**a–c**) but for sulfate aerosols (ng•m^−3^). Wind vectors in (**d–f,i**) are obtained from model simulations. The vertical velocity field has been scaled by 300 m•s^−1^ (Figure created using the COLA/GrADS software).

**Figure 4:**
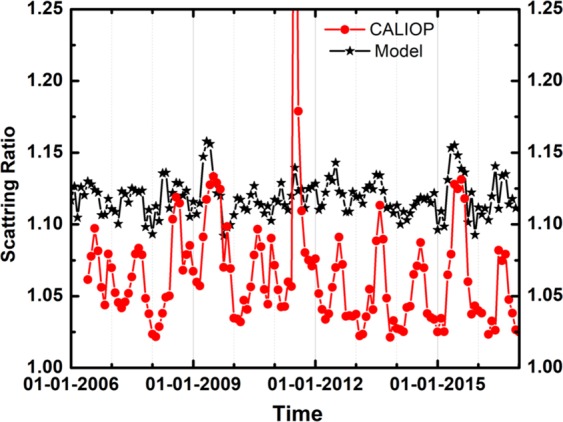
Time series of Scattering Ratio (SR) averaged for 15–18 km over the Indo-Gangetic plain and Tibetan Plateau region (25–35°N, 70–100°E) from the ECHAM5-HAMMOZ aeronAMIP simulation (black) and CALIOP (red). (Figure created using the Origin (OriginLab, Northampton, MA)).

We utilize the ECHAM5-HAMMOZ state-of-the-art aerosol-chemistry-climate model (see Methods and experiment details in Table 1) in order to understand the formation and possible impact of this elevated aerosol over the Indian monsoon region. Our model simulations show the aerosol layer (ATAL) during the monsoon season over South Asia (23–40°E) and its anomalous enhancement during recent El Niño events (Fig. 3a–c). The higher aerosol loading in the ATAL (greater aerosol extinction by 3E-4 km^−1^ to 5E-4 km^−1^ near 100 hPa) strengthens the layer over the entire North-Indian belt, which is mainly governed by the vertical transport of aerosols from East Asia (~110–120°E). Model simulations for the El Niño years 2009 and 2015 also show higher aerosol loading (3E-4 km^−1^ to 5E-4 km^−1^) in the ATAL (Fig. S1a,b). This enhancement during the monsoons season of El Niño years 2009 and 2015 is also evident in the scattering ratio over North India and Tibetan Plateau (70–100°E, 25–35°N) from CALIOP and model simulation (Fig. 4). Despite the influence of the Sarychev volcanic plume entrained on the periphery of the monsoon anticyclone, the air inside the anticyclone over North India remained relatively isolated^11,36^, with the peak observed on CALIOP measurements during the summer 2009 mainly reflecting the influence of El Niño. It has to be mentioned here that the excess aerosol loading in ATAL is visible in the years with a persistent El Niño condition throughout the summer monsoon season (JJAS). The year 2014 was likewise an El Niño year with considerable precipitation deficiency in June and July associated with the warm Pacific SST anomaly, which eventually turns out to be less-deficit/moderate-excess in the following months (August/September) accompanied with the decrease warming in Pacific SST^37^. Due to the changes in the circulation pattern, especially in the second half of the season, the aerosol uplifting from the east Asian region becomes relatively weak (Fig. S2), resulting in smaller aerosol extinction values in 2014 compared to 2015 (Fig. 4).

**Table 1:** Details of experiments conducted in the present study.

Sr No.	Name of the Experiment	Sea surface temperature (sst)	Period of simulations	Aerosol switched on/off	Experiment description
1	aeroffCL	Climatological SST	Ensemble since 21May 31May 2003	off	Passively transported aerosol (aero-off)- with Climatological SST
2	aeronCL	Climatological SST	Ensemble since 21May 31May 2003	On	Interactive aerosols (aero-on) with Climatological SST
3	aeroffEL	Canonical El Niño type SST (Fadnavis *et al*., 2017)	Ensemble since 21May 31May 2003	off	Passively transported aerosol (aero-off) with El Niño SST
4	aeronEL	Canonical El Niño type SST (Fadnavis *et al*., 2017)	Ensemble since 21May 31May 2003	on	Interactive aerosols (aero-on) with El Niño SST
5	aeronAMIP	Monthly varying AMIP SST	January 2005-December 2016	on	Interactive aerosols with monthly varying AMIP SST and forced with meteorology
6	aeroffAMIP	Monthly varying AMIP SST	January 2005-December 2016	off	Passively transported aerosol (aero-off) with monthly varying AMIP SST and forced with meteorology

During El Niño, a robust vertical ascent is visible over East Asia, which can lift boundary layer pollutants from East Asia into the UTLS region (Fig. S3). These pollutants are further transported to South Asia from East Asia. At the same time, the ascent over 90–100°E further promotes the vertical transport from South Asia along with the strong ascent over East Asia (Fig. S3b). Increased aerosols in the El Niño simulations (aeronEL) clearly show the strong ascent over East Asia (~110–120°E in the troposphere; and 90–100°E in the upper troposphere 500–150 hPa) (Fig. S3d). The strong ascent along with the rising branch of circulation from East Asia further enriches the aerosols in the UTLS over South Asia and therefore promoting the enhanced subsidence over Indian region (70–90°E).

Aircraft, balloon sonde measurements and model simulations suggest that the aerosols in the ATAL is composed of organic carbon, black carbon, nitrate and sulfate aerosols^12,15,17,23^. Lofting of black carbon aerosols (mostly absorbing) from North India and East China accumulates during El Niño, while sulfate aerosols (mostly scattering) are transported from East-Asia (Fig. 3d–i). Figure 3 illustrates how the aerosol enters the ATAL mainly from East Asia during El Niño. The black carbon and sulfate aerosol loadings are large during El Niño over the southern slopes of the Himalayas and Tibetan Plateau (TP) (85°E–100°E) (1000–300 hPa), and in the ATAL (130–70 hPa).

**Figure 5:**
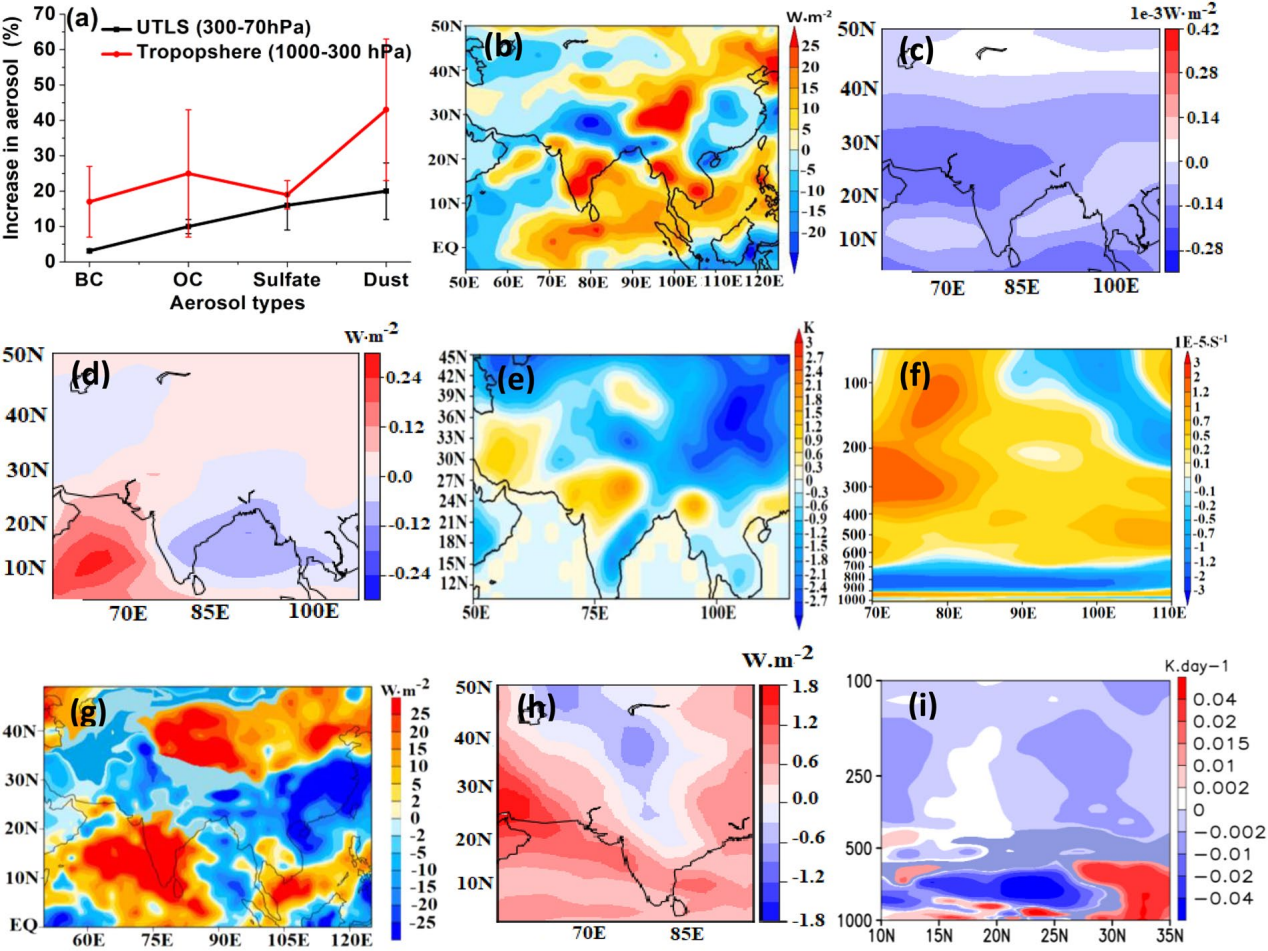
(**a**) Percentage increase in UTLS (300–70 hPa) and troposphere (1000–300 hPa) aerosols during El Niño at a location in the ATAL (30°N, 90°E). (**b**) aerosol induced simulated changes in solar surface solar radiation (W•m^−2^) (aeronEL-aeronCL). Simulated changes in surface solar radiation (mW.m^−2^) due to aerosols in (**c**) the UTLS (300–70 hPa) (aeronEL-aeronCL), and (**d**) for the troposphere (1000–300 hPa) (aeronEL-aeronCL). (**e**) aerosol-induced anomalies in surface temperature (K) (aeronEL-aeronCL), (**f**) aerosol induced anomalies of Brunt Väisäla frequency (1E-5 sec^−1^) during El Niño (aeronEL-aeroffEL) (ave:20–35°N). Simulated changes in surface radiation (W•m^−2^) due to (**g**) all 52 gas phase species in ECHAM (aeroffEL-aeroffCL), (**h**) UTLS methane, water vapour and ozone in the offline model. (**i**) Net heating rates (ave: 20–35°N) from aerosols and gases (52 gas phase species) in ECHAM model simulations (K•day^−1^) (aeroffEL-aeroffCL). (Figure created using the COLA/GrADS software).

Figure 5a shows the percentage increase of each type of aerosol in the troposphere (1000–300 hPa) and in the UTLS (300–70 hPa) during El Niño. The enhancement of sulfate, dust, and organic carbon aerosols in the UTLS thickens and widens the ATAL over the South Asian region. Additionally, the higher amounts of sulfate aerosols in the UTLS can significantly cool the surface by scattering incoming solar radiation^38^. Figure 5b illustrates the aerosol-induced reduction in solar radiation at the surface during El Niño (aeronEL - aeronCL), with simulated values of −5 to −20 W•m^−2^ over northern India (70–90°E; 25–35°N). While this effect is caused by the aerosol enhancement during El Niño in the free troposphere and UTLS, there is also a considerable contribution from UTLS aerosol changes alone. Using dedicated model experiments, we quantify the effect on solar radiation at the surface due to aerosol changes during El Niño (aeronEL-aeronCL) in both these regions (Fig. 5c,d). The presence of higher aerosol in the UTLS during El Niño leads to a reduction in solar radiation at the surface over Asia, with a minimum of −0.25 mWm^−2^ over the Bay of Bengal. The tropospheric aerosol changes during El Niño, lead to surface solar radiation increases (0.25 W•m^−2^) over the Arabian Sea, north India, and the northern part of East Asia and decreases (−0.25 W•m^−2^) over the Indian peninsula, Bay of Bengal and the southern part of East Asia. The inhibition of surface solar radiation by the aerosol layer during El Niño has a cooling effect at the surface. The model simulations show an associated surface cooling of 1.5 K to 2 K at most locations except the desert regions of west-Asia and Mongolia (Fig. 5e), therefore increasing the stability of the troposphere (Fig. 5f). It should be mentioned that, in addition to the role of the enhanced aerosol layer quantified here, other El Niño induced changes in the UTLS region (i.e. changes in, distribution of greenhouse gases like water vapour, ozone or methane)^39^ will also have important associated radiative effects. By contrasting two sensitivity simulations (aeroffEL-aeroffCL), we find that the change in UTLS gases (for 52 gas phase species^40^) also reduces surface solar radiation (−5 to −15 W•m^−2^) over North India, comparable to the effect of aerosols (−10 to −20 W•m^−2^) (Fig. 5b,g). Using offline radiative calculations^41,42^, we estimate that the increase in UTLS ozone, methane and water vapour during El Niño (aeronEL-aeronCL) leads to mean regional (20–40°N, 75–85°E) surface solar radiation changes of −0.6 to −1.1 W•m^−2^. This suggests that the changes in gases in the UTLS will contribute alongside the aerosol-induced radiative effects at the surface (Fig. 5h). Also, the combined heating rates changes from aerosols and gases produce significant cooling in the mid-upper troposphere over North India and Tibetan Plateau region (20–35°N) (Fig. 5i), which promote subsidence over this region.

### The aerosol layer and intensifying monsoon droughts

**Figure 6:**
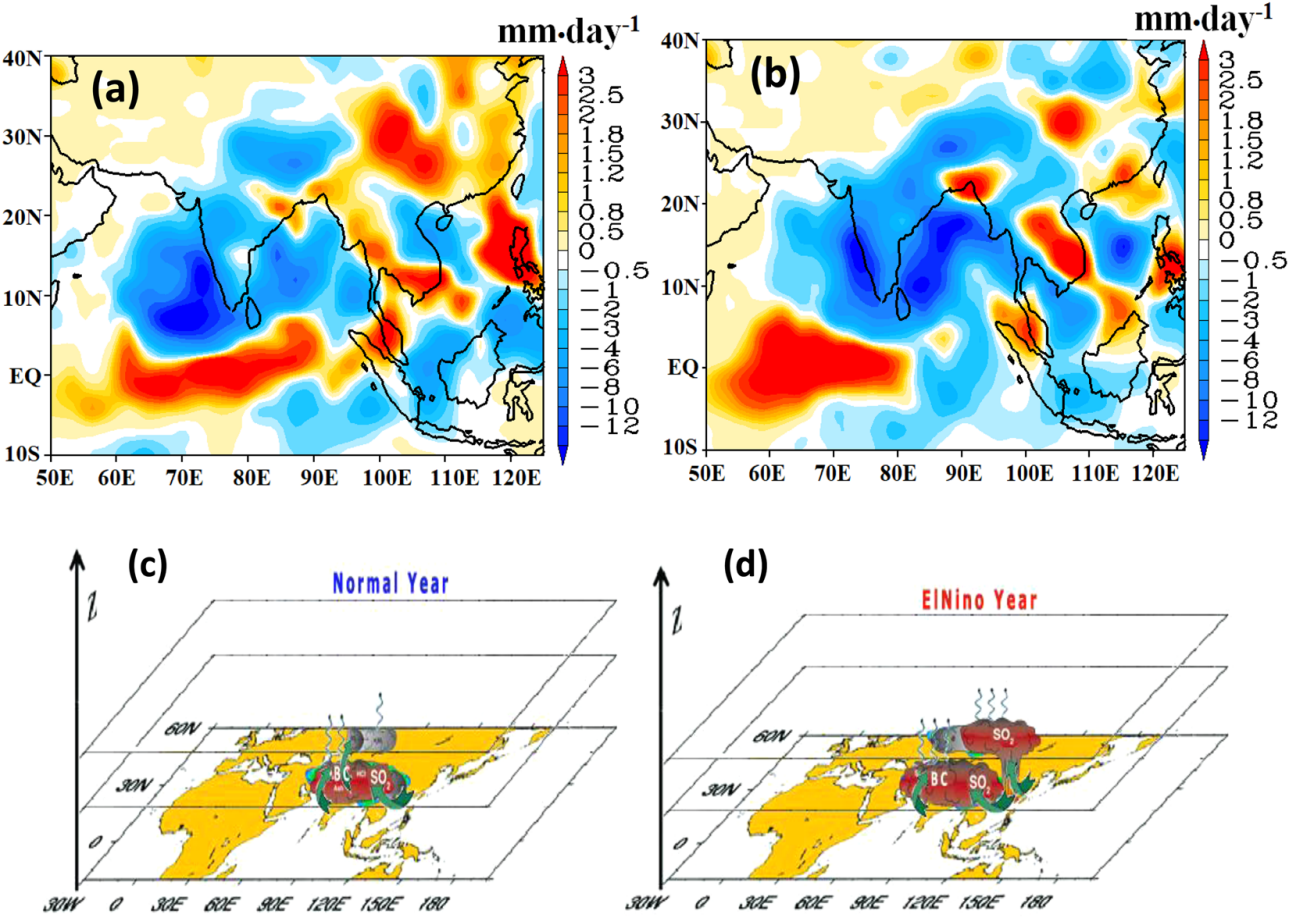
Seasonal mean (July - September) precipitation anomalies (mm•day^−1^) obtained from (**a**) aeroffEL-aeroffCL simulation, indicating reduced rainfall during El Niño than climatology when no aerosols were introduced, (**b**) aeronEL-aeronCL simulation, indicating reduced rainfall during El Niño relative to climatology when aerosols were introduced. Comparison of **a,b** indicates that aerosols have increased the scarcity of drought in El Niño simulation. A schematic depicts aerosol distribution in the ATAL during (**c**) normal and (**d**) co-occurring El Niño simulation. Vertical transport of sulfate aerosols from East Asian during El Niño is shown in (**d**). (**a,b** created using the COLA/GrADS software and **c,d**) plotted using NCL and 3D effect and schematic designs created using Adobe illustrator).

The El Niño itself leads to a decrease in rainfall over India (Fig. 6a), with a suppressed rainfall during the summer season estimated at 2 to 6 mm•day^−1^. The inclusion of aerosols (aeronEL-aeronCL) amplifies decrease in rainfall by 17% over the central India (10–25°N, 70–85°E). We estimate (aeronAMIP-aeroffAMIP experiments) that the aerosol loading in 2009 and 2015 has induced a rainfall deficit of 1–7 mm•day^−1^ (i.e. ~14%) over the central India (Fig. S4a,b). The combined effect of El Niño and the increased aerosol loading leads to a rainfall deficit of approximately 4 to 12 mm•day^−1^ over India (Fig. 6b). A schematic depicting aerosol distribution in the ATAL during normal and co-occurring El Niño simulation is shown in Fig. 6c,d.

This combined effect produces an aggravated subsidence over the Indian region compared to the individual effect of El Niño. The effect of aerosols weakens the zonal winds and enhances the anomalous subsidence over the monsoon region from the meridional overturning circulation over 15–30°N (Figs S3, S5a,b). The upper tropospheric temperature has a significant role in driving the monsoon circulation from the ocean to the sub-continent^43,44^. The strong cooling during El Niño (Fig. S5c) is further intensified by the presence of the aerosols (Fig. S5d). This weakens the zonal inflow from the ocean, reducing the strength of a feeble monsoon Hadley circulation^2^, which in turn leads to an anomalous large-scale subsidence and suppresses the convection, therefore enhancing the severity of droughts^1,2^. The anomalous cooling over North India and TP is a consequence of the enhanced aerosol loading over the South Asian upper troposphere. While the general overestimation of UTLS aerosols in our model suggests that the impact on the severity of droughts is likely smaller than estimated here, this might be balanced by the additional contribution from nitrate aerosols^45^ (not simulated here).

We attribute the increase of drought severity to the following mechanism:

1. The El Niño induced atmospheric circulation lifts the boundary layer aerosol from East Asia to the UTLS and then to West Asia into the ATAL region.
2. The added blanket of aerosol reduces the solar insolation, cooling the upper troposphere over the Tibetan Plateau and surface over North India.
3. The resulting enhanced stability leads to a weakening of the monsoon Hadley circulation.
4. The anomalous large-scale subsidence results in amplifying the severity of monsoon droughts.
5. There are additional radiative impacts from changes in UTLS gases also contributing to the increase in monsoon droughts severity.

## Summary and Discussion

The ECHAM5-HAMMOZ is utilised to understand the role of the elevated aerosol layer in exacerbating the severity of monsoon droughts during El Niño. The observed changes can be attributed to the changes in aerosol fields. The increase in industrial emissions from India and China adds millions of metric tons of aerosol into the atmosphere^35,46^. In a global warming environment, the tropical Pacific is predicted to witness more frequent extreme El Niño events^47–49^. Even if we are to follow the more moderate RCP4.5 IPCC scenario^50^, aerosol loadings over South Asia are still expected to remain large at least until the end of the 2040 s. Thus, the more extreme El Niño events, in a background of rising aerosol emission will have an adverse impact on the severity of droughts over the monsoon region. Another important aspect is that the distinct role of aerosols during the pre-monsoon and the monsoon season in the presence of El Niño. While pre-monsoon aerosol loading over North India and the TP reduces the severity of drought^33^, aerosol increases during the monsoon enhance it.

While highlighting the complex relationship between droughts, El Niño and aerosols, the present study addresses the potential consequences of simultaneous occurrence of multiple adverse climate drivers (El Niño and aerosols) which may amplify the severity of meteorological phenomena in the present day climate. The plains of North–Central India are under hydrological stress^51^ and already exposed to the vulnerability of hydrological extremes^52^. The increase in the severity of droughts accompanied with El Niño has a profound societal impact that can dry the whole of Central India, affecting agriculture and the livelihood of millions of people. It is well known that reducing aerosol emissions is essential from an air quality perspective, but here we highlight its additional effects on the regional monsoon hydro-climate. These two benefits of decreased pollution levels would promise a better future and would help to avoid negative consequences for millions of lives.

## Methods

### Model description and experimental setup

We perform sensitivity experiments using state of the art ECHAM5-HAMMOZ aerosol-chemistry-climate model. The model comprises an atmospheric general circulation module, ECHAM5^53^, a tropospheric chemistry module, MOZ^54^, and an aerosol module, namely the Hamburg Aerosol Model (HAM)^55^. The model parameterization and other details are previously documented^13,56^. The model set-up consists of nitrates in gaseous form and not in the particulate aerosol speciation. A cirrus cloud scheme has been previously described^57,58^.

The model simulations were performed at spectral resolution T42 corresponding to 2.8° × 2.8° in horizontal and 31 vertical levels from surface to 10 hPa with a time step of 20 minutes. To avoid the effect of pre-monsoon aerosols, we started the model integration from 21 May to 31 May to generate ten ensemble members. The model was spun up for one month, and analysis is presented for the monsoon season (July–September). The simulations were performed for the year 2003 since it was a normal monsoon rainfall year and there was no Indian Ocean Dipole (IOD) or El Niño. The anthropogenic and biomass burning emissions are from the year 2000 RETRO project dataset (available at http://eccad.sedoo.fr/)^59^. The sulfate, black carbon and organic carbon emission are from AEROCOM-II emission inventory for the year 2000^60^.

In four sets of experiments, namely, aeronCL, aeroffCL, aeronEL and aeroffEL the model is forced with a monthly varying climatological SST as a lower boundary condition. The monthly climatological SST is derived from HadISST (Hadley Centre Global Sea Ice and Sea Surface Temperature) for the period 1979–2010. For the aeronEL, and aeroffEL, El Niño experiments, the model is forced with SSTs typical of an El Niño (Table 1). Specifically, the SSTs for the El Niño experiments are obtained by imposing monthly SST anomalies of 1997 (for April-September) only in the tropical Pacific region (110–90 °W, 20 °S–20°N) on climatological SSTs (see Fig. S6 from previous study^33^). In aeronAMIP and aeroffAMIP experiments (2005–2016) the model is forced with monthly varying SST and meteorology.

### Observations and reanalysis data

Aerosol distribution during the monsoon season is studied from three different satellites (1) Aerosol Index from Total Ozone Mapping Spectrometer (TOMS) for July-September during the period 1979–2005 (https://disc.gsfc.nasa.gov/datasets/TOMSEPL3_V008/summary), (2) Aerosol Optical Depth (AOD) from Multi-Angle Imaging Spectroradiometer (MISR)^61^ for July-September during the period 2000–2013 (https://misr.jpl.nasa.gov/getData/accessData/), (3) CALIOP Scattering Ratio (SR) for July - August during June 2006–December 2015 (https://eosweb.larc.nasa.gov/project/calipso/calipso_table). The TOMS detects aerosols over bright targets (e.g. deserts), while MISR provides aerosol information over the oceans and land^62,63^. TOMS aerosol index data during 1979–2005 comprises measurements from two different instruments Nimbus-7 (Nov 1978–May 1993) and Earth Probe (July 1996–2005). The TOMS AI data after 2001 is not recommended for trend analysis due to calibration issues associated with sensor degradation (http://daac.gsfc.nasa.gov/guides/GSFC/guide/tomsl3 dataset.gd.shtml)^64^. However, this data is considered in the current study since the focus is to study the seasonal variation.

The CALIOP lidar makes range-resolved measurements of elastic backscatter at 532 and 1064 nm and of linear depolarization ratios at 532 nm^65^. In this study, we analyse CALIOP lidar measurements of total backscatter at 532 nm and depolarization ratios at 532 nm. Due to the low signal-to-noise ratio of the CALIOP signal in the UTLS, a specific treatment of the level 1 data was developed to infer backscatter profiles from the upper troposphere to 40 km^34^. This technique uses a depolarization ratio threshold of 5% to remove clouds, horizontal averaging of ~100 km to increase SNR and is similar to what has been previously published^11,12,15,34^. It reveals the presence of the ATAL during every summer Asian monsoon period, even in the absence of a volcanic eruption, confirming earlier results^11,12^. This is also verified by the stratospheric aerosol product developed by the CALIPSO team^66^. The ATAL has also been detected by the Stratospheric Aerosol and Gas Experiment (SAGE) II data between the late 90’s to 2005^12^. The stratospheric aerosol product dataset is now publically^34^ available at https://eosweb.larc.nasa.gov/project/calipso/stratospheric_table.

We used zonal and meridional wind data from National Center for Environmental Prediction (NCEP) reanalysis, available for the period 1948–2009^67^ the rainfall data from GPCP (Global Precipitation Climatology Project) for the period 1979–2015 (https://www.esrl.noaa.gov/psd/data/gridded/data.gpcp.html) and TRMM_3B42 (version 7) rainfall estimate for the period 1997–2016 (https://pmm.nasa.gov/data-access/downloads/trmm). In this study, we have considered only strong El Niño years 1957, 1965, 1967, 1972, 1977, 1982, 1991, 1994, 1997, 2002, 2004, 2009 and 2015 when the amplitude of the SST anomalies in either eastern or central tropical Pacific exceed one standard deviation. Also, the Niño-3 index must be more than the threshold value (>0.5) from June to September. In our analysis, we considered monsoon season of the co-occurring El Niño year since lead-lag correlations between all Indian summer monsoon rainfall and Niño-3 index exhibits a maximum negative correlation for zero lag year (details are documented previously)^33,68^

### Radiative forcing calculations

The aerosol direct radiative forcing (DRF) in the aeronCL and the aeronEL simulations and the radiative forcing of methane, ozone and water vapour were calculated using the offline version of the SOCRATES radiative transfer model^41,42^ with six shortwave and nine longwave bands, and a delta-Eddington two-stream scattering solver at all wavelengths. Aerosol scattering and absorption coefficients, together with asymmetry parameters, were calculated for each aerosol size mode and spectral band^69^. The indirect aerosol effect was not considered here. We also compared the relative radiative forcing in the lower-mid troposphere and in the UTLS by performing additional simulations where changes in aerosols, methane, ozone and water vapour were only applied at 1000–300 hPa and at 300–70 hPa, respectively.

### Model evaluation

In spite of the fact that the spatial variations of the scattering ratio reproduced reasonably well (Figs 1 and 4), the model overestimated its magnitude. This can be due to various factors such as uncertainties in (i) aerosol emission inventories, (ii) model representation of transport and chemistry processes, or (iii) satellite scattering ratio measurements (e.g. cloud filtering). Comparison of simulated AOD with MISR observations (Figs 2 and S6) indicates that the model under-estimates AOD (~0.1) over the Thar desert, Iran, Turkmenistan and Kyrgyzstan and overestimates (~0.3) over the Arabian Sea. Figure S7a,b shows seasonal mean rainfall for climatology and El Niño simulations. The model reproduces the El Niño related deficit rainfall: the comparison of simulated Niño related rainfall anomalies over the Indian region indicates that spatial pattern of simulated rainfall is in reasonable agreement with GPCP and TRMM observations, although simulated rainfall is underestimated in the model (~4–6 mm•day^−1^, see Fig. S7a–e).

### Transport of aerosols in the lower troposphere

The distribution of black carbon and dust aerosols in the troposphere (860 hPa) and mid-troposphere (500 hPa) from aeronCL and aeronEL simulations is illustrated in Fig. S8. We mention that the distribution of organic carbon and sulfate is similar to that of black carbon. The model simulations show higher amounts of these aerosols over the Indo-Gangetic Plains in the lower troposphere for El Niño simulations than climatology (Fig. S8a–h). The dust transported from west Asia accumulates over north India and the TP^70^. It is known that there is a reduced rainout during El Niño^71^. The tropospheric aerosol column also contributes to an increasing scarcity of rainfall over India. It should be noted that aerosols in the troposphere have an important influence on precipitation by modifying cloud microphysics over shorter timescales. previous study^10^ has shown that, over the timescale of a week, the rainfall over central India (16.5–26.5°N, 74.5–86.6°E) is positively correlated with the concentration of natural aerosols such as desert dust and sea salt over the Arabian Sea. All these further indicate the importance and complexity of aerosols in modulating the Indian monsoon.

## Supplementary information

Elevated aerosol layer over South Asia worsens the Indian droughts
