# Supplementary material for: Elevated aerosol layer over South Asia worsens the Indian droughts
Source: Sci Rep. 2019 Jul 16;9:10268. doi: 10.1038/s41598-019-46704-9 (PMC6635485; doi:10.1038/s41598-019-46704-9)
Supplement: Supplementary file 1 — Elevated aerosol layer over South Asia worsens the Indian droughts [file 41598_2019_46704_MOESM1_ESM.pdf]

Elevated aerosol layer over South Asia worsens the Indian droughts

Suvarna Fadnavis[1], T.P. Sabin[1], Chaitri Roy[1], Matthew Rowlinson[2], Alexandru Rap[2], Jean-Paul Vernier[3, 4], Christopher E. Sioris[5]

[1]Indian Institute of Tropical Meteorology, Pune, India

[2]School of Earth and Environment, University of Leeds, Leeds, UK.

[3]National Institute of Aerospace, Hampton, Virginia

[4]NASA Langley Research Center, Hampton, Virginia

[5]Air Quality Research Division, Environment and Climate Change, Toronto, Canada

Corresponding author: suvarna@tropmet.res.in

Supplementary figures

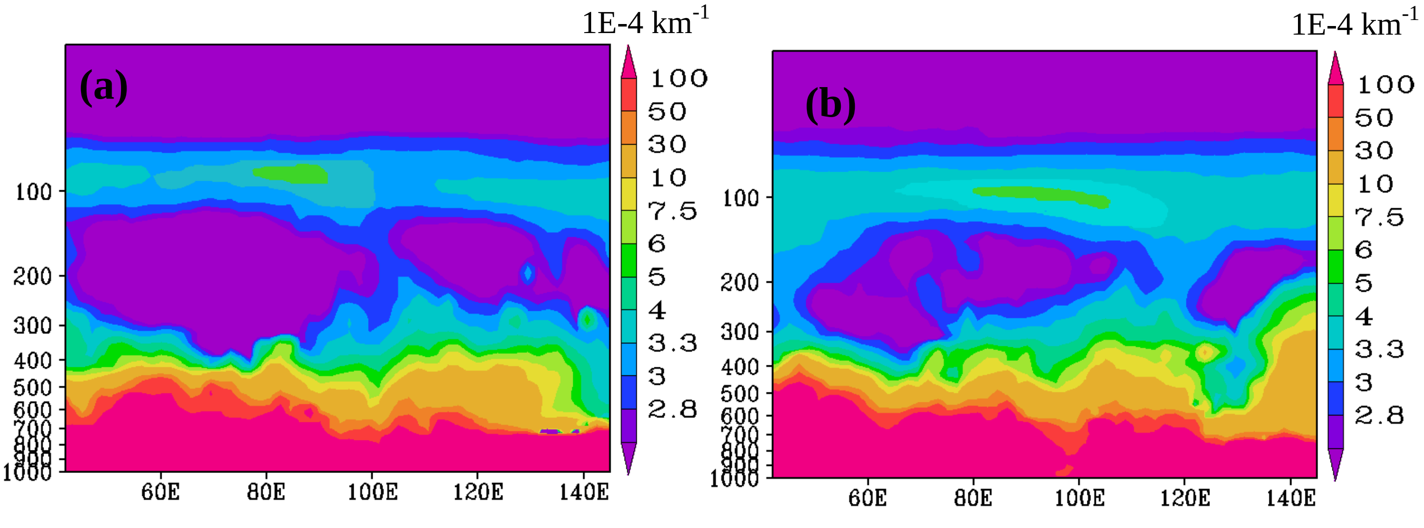

Figure S1: Zonal cross section (averaged for July – September and 23 – 40 °N) of aerosol extinction (1E-4 km^{-1}) as obtained from aeronAMIP simulations for the year (a) 2009, (b) 2015. [Figure created using the COLA/GrADS software].

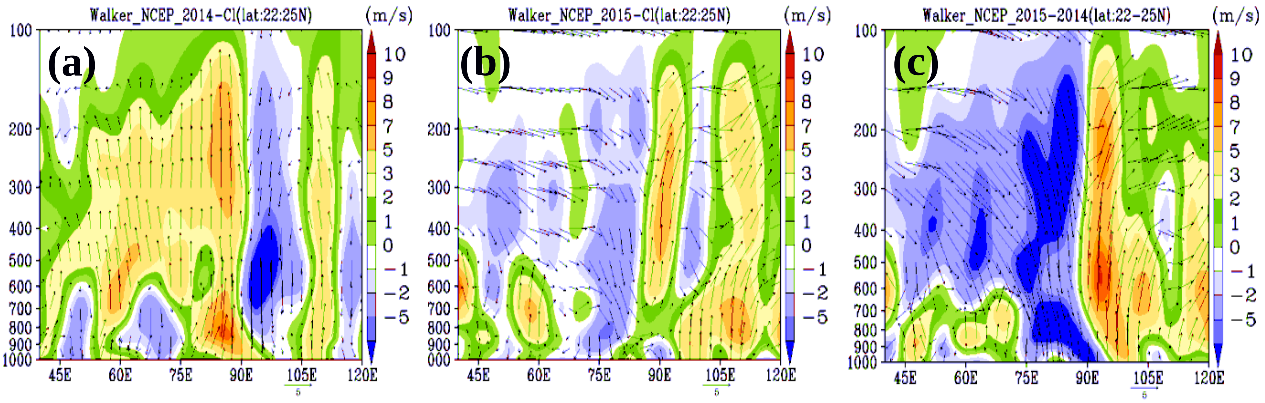

Figure S2: Cross-section of anomalies of seasonal mean zonal circulation (m•s^{-1}) from NCEP reanalysis, averaged over 22 – 25°N for (a) 2014, (b) 2015, (c) difference between (a) and (b). Climatology removed from the respective year is based on the data from 1948 to 2007.

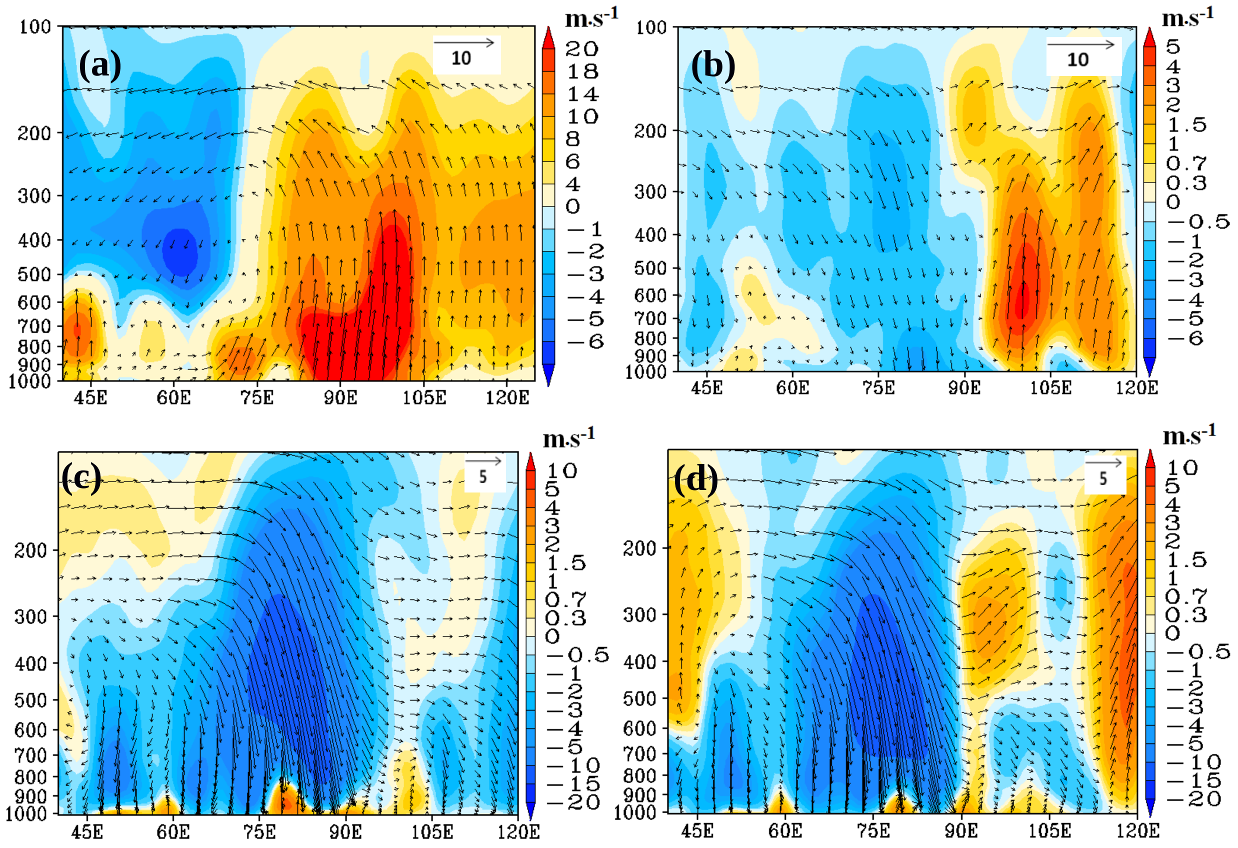

Figure S3: Cross-section of seasonal mean zonal circulation (m•s^{-1}) averaged over 15 – 30 °N for (a) NCEP reanalysis climatology (1948 – 2007), (b) NCEP reanalysis El Niño years anomalies, and simulated (c) aeroffEL- aeroffCL, and (d) aeronEL-aeronCL anomalies. The coloured contours show the vertical velocity field scaled by 300 m•s^{-1}. [Figure created using the COLA/GrADS software].

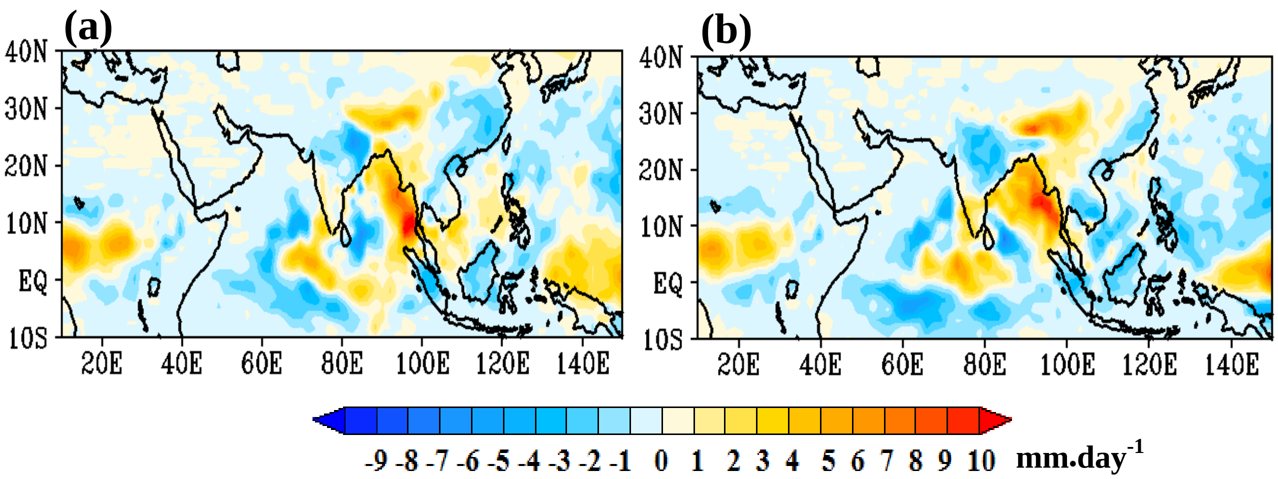

Figure S4: Simulated (aeronAMIP-aeroffAMIP) seasonal mean (July - September) precipitation anomalies (mm•day^{-1}) for (a) 2009, (b) 2015. [Figure created using the COLA/GrADS software].

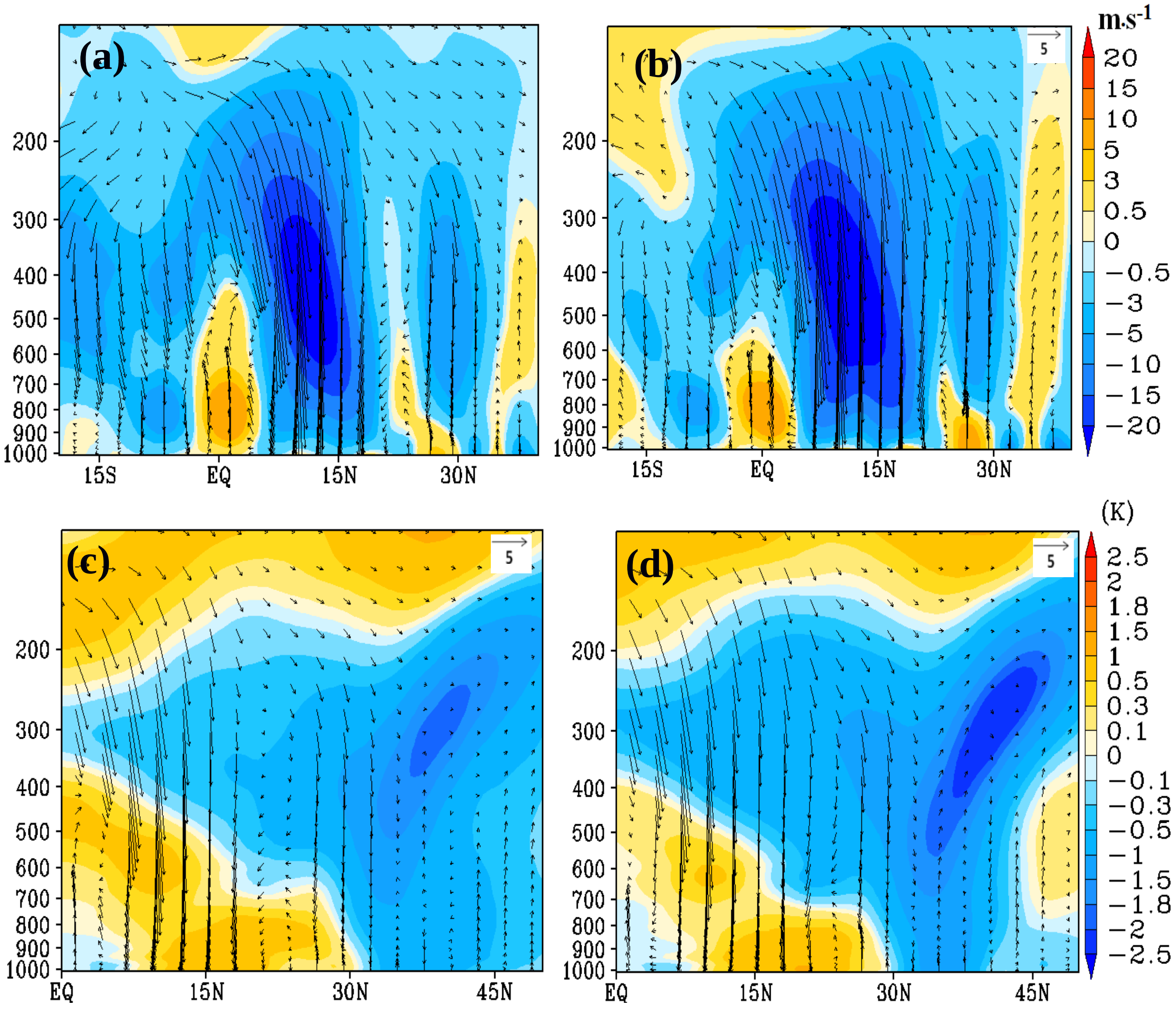

Figure S5: Simulated meridional cross sections averaged for 70 – 100°E and July-September of aerosol-induced changes in from (a) circulation (m•s^{-1}) from aeroffEL- aeroffCL, (b) circulation (m•s^{-1}) from aeronEL- aeronCL (c) temperature (K) from aeroffEL- aeroffCL and (d) temperature (K) from aeronEL- aeronCL. The contours indicate the vertical velocity field scaled by 300 m•s^{-1}. [Figure created using the COLA/GrADS software].

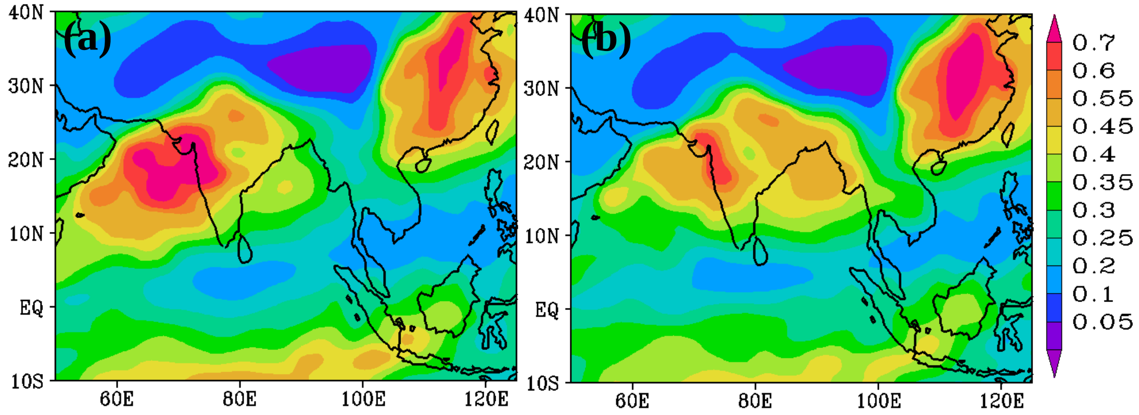

Figure S6: Simulated (a) aeronCL and (b) aeronEL aerosol optical depth at 550 nm. [Figure created using the COLA/GrADS software].

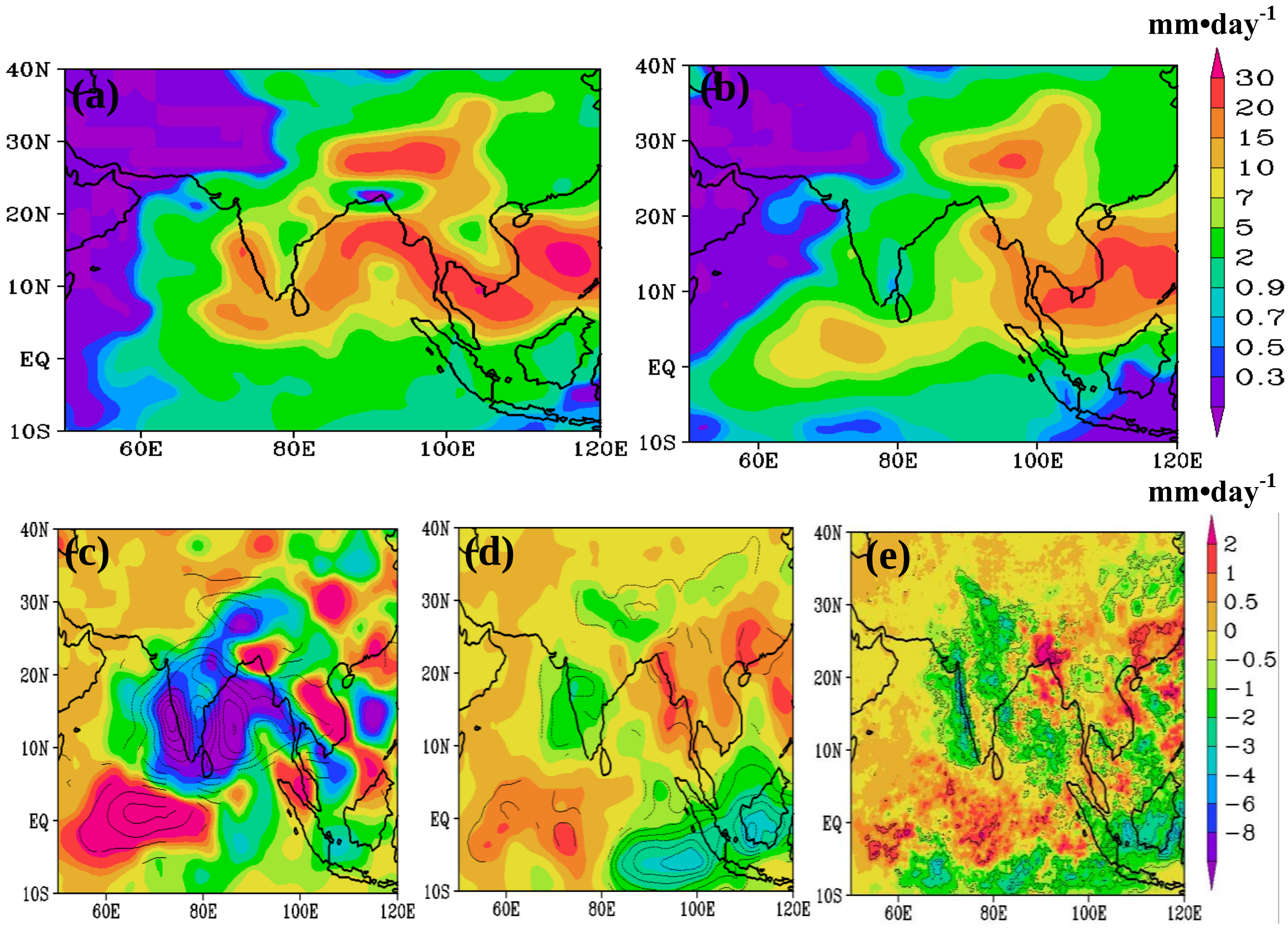

Figure S7: Spatial distribution of simulated rainfall (mm•day^{-1}) for (a) aeroffCL, (b) aeroffEL, (C) aeroffEL-aeroffCL, (d) composite of difference between El Niño years and climatology from GPCP, (e) composite of difference between El Niño years and climatology from TRMM. Contour lines in (c)-(e) indicate 99% significance levels. [Figure created using the COLA/GrADS software].

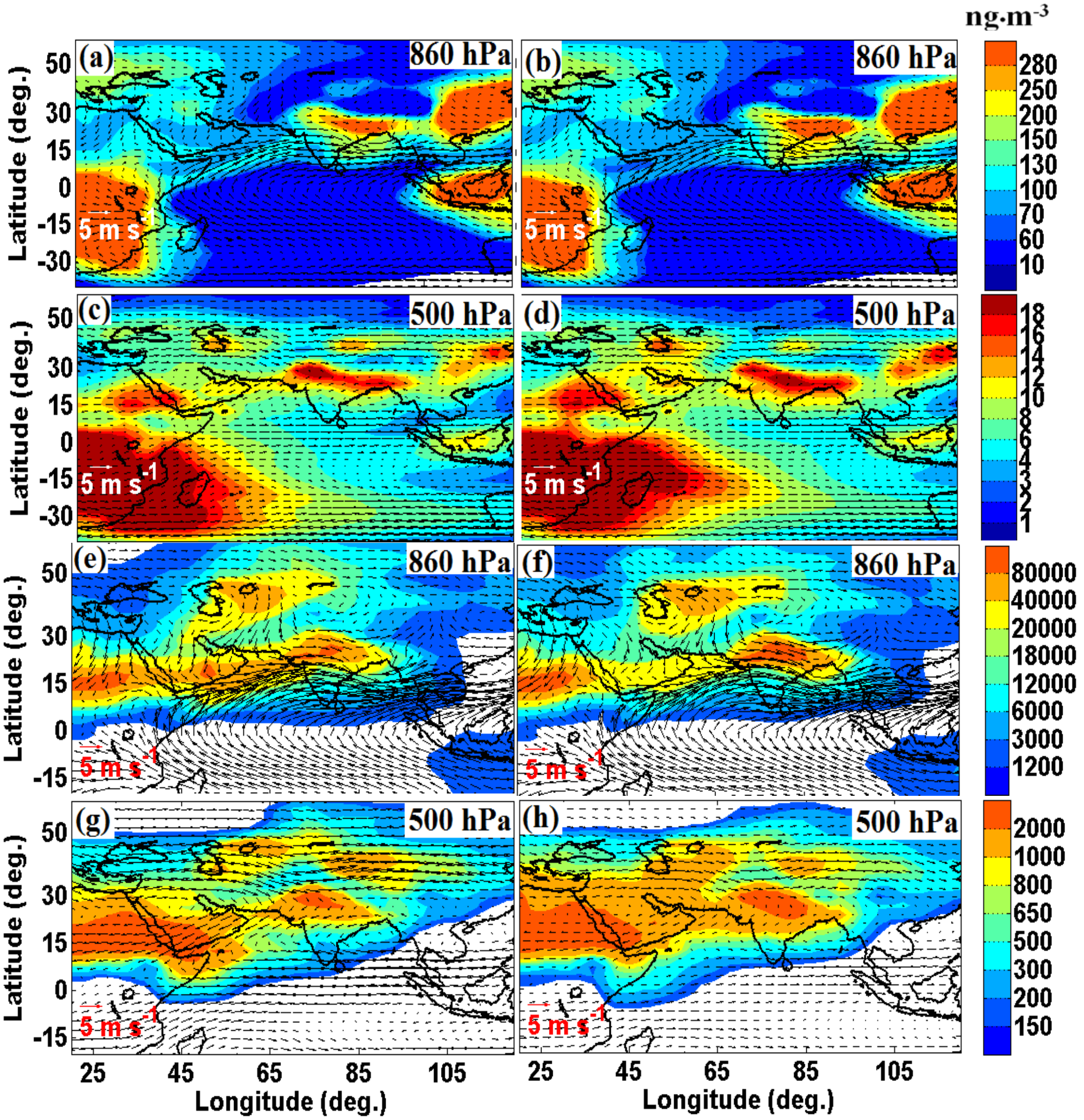

Figure S8: Simulated summer mean aerosol loadings (ng•m^{-3}): (a) BC at 860 hPa in aeronCL, (b) BC at 860 hPa in aeronEL, (c) BC at 500 hPa in aeronCL, (d) BC at 500 hPa in aeronEL, (e) dust at 860 hPa in aeronCL, (f) dust at 860 hPa in aeronEL, (g) dust at 500 hPa in aeronCL, (h) dust at 500 hPa in aeronEL. Wind vectors are indicated with black arrows. [Figure created using MATLAB software].
